# Biocide-Induced Emergence of Antibiotic Resistance in *Escherichia coli*

**DOI:** 10.3389/fmicb.2021.640923

**Published:** 2021-02-26

**Authors:** Beatriz Merchel Piovesan Pereira, Xiaokang Wang, Ilias Tagkopoulos

**Affiliations:** ^1^Microbiology Graduate Group, University of California, Davis, Davis, CA, United States; ^2^Genome Center, University of California, Davis, Davis, CA, United States; ^3^Department of Computer Science, University of California, Davis, Davis, CA, United States

**Keywords:** evolution, *E. coli*, antiseptics, disinfectants, antibiotic resistance

## Abstract

Biocide use is essential and ubiquitous, exposing microbes to sub-inhibitory concentrations of antiseptics, disinfectants, and preservatives. This can lead to the emergence of biocide resistance, and more importantly, potential cross-resistance to antibiotics, although the degree, frequency, and mechanisms that give rise to this phenomenon are still unclear. Here, we systematically performed adaptive laboratory evolution of the gut bacteria *Escherichia coli* in the presence of sub-inhibitory, constant concentrations of ten widespread biocides. Our results show that 17 out of 40 evolved strains (43%) also decreased the susceptibility to medically relevant antibiotics. Through whole-genome sequencing, we identified mutations related to multidrug efflux proteins (*mdfA* and *acrR*), porins (*envZ* and *ompR*), and RNA polymerase (*rpoA* and *rpoBC*), as mechanisms behind the resulting (cross)resistance. We also report an association of several genes (*yeaW*, *pyrE*, *yqhC*, *aes*, *pgpA*, and *yeeP*-*isrC*) and specific mutations that induce cross-resistance, verified through mutation repairs. A greater capacity for biofilm formation with respect to the parent strain was also a common feature in 11 out of 17 (65%) cross-resistant strains. Evolution in the biocides chlorophene, benzalkonium chloride, glutaraldehyde, and chlorhexidine had the most impact in antibiotic susceptibility, while hydrogen peroxide and povidone-iodine the least. No cross-resistance to antibiotics was observed for isopropanol, ethanol, sodium hypochlorite, and peracetic acid. This work reinforces the link between exposure to biocides and the potential for cross-resistance to antibiotics, presents evidence on the underlying mechanisms of action, and provides a prioritized list of biocides that are of greater concern for public safety from the perspective of antibiotic resistance.

**Significance Statement:**

Bacterial resistance and decreased susceptibility to antimicrobials is of utmost concern. There is evidence that improper biocide (antiseptic and disinfectant) use and discard may select for bacteria cross-resistant to antibiotics. Understanding the cross-resistance emergence and the risks associated with each of those chemicals is relevant for proper applications and recommendations. Our work establishes that not all biocides are equal when it comes to their risk of inducing antibiotic resistance; it provides evidence on the mechanisms of cross-resistance and a risk assessment of the biocides concerning antibiotic resistance under residual sub-inhibitory concentrations.

## Introduction

Biocides are chemicals which include antiseptics and disinfectants, used to control, eliminate, or reduce the number of undesired organisms (Scenihr and European Commission, 2009). Despite their ubiquitous presence, the evolution of biocide resistance is much less studied than that in antibiotics. Whether the excessive and often unregulated use of disinfectants and antiseptics leads to biocide tolerance or cross-resistance to antibiotics remains a controversial topic, with voices both for Sidhu et al. (2002), Gilbert and McBain (2003), Condell et al. (2012), Webber et al. (2015), Bhardwaj et al. (2016), Mulder et al. (2018) and against (Gilbert and McBain, 2003; Gerba, 2015) its support. Concomitantly, the mechanisms of action of biocide adaptation and resistance are generally unknown (Morente et al., 2013). A few exceptions exist, such as the chemical triclosan, recently banned in the United States (Food and Drug Administration, 2016), for which the mechanisms of adaptation and resistance have been thoroughly described (Heath et al., 1999; Webber et al., 2008).

How resistance emerges is usually studied in controlled environments through adaptive laboratory evolution (ALE). ALE studies have been conducted in *Escherichia coli* (*E. coli*) for a range of stressful conditions, which included acid (Johnson et al., 2014; Harden et al., 2015; Zorraquino et al., 2017), osmotic (Zorraquino et al., 2017), antibiotics (Lázár et al., 2014; Oz et al., 2014), and biocides (Braoudaki and Hilton, 2004; Bore et al., 2007; Wand et al., 2017; Abdelaziz et al., 2019; Verspecht et al., 2019). The adaptation to common disinfectants and antiseptics, and the emergence of cross-resistance to chemicals such as antibiotics, was demonstrated for bacteria exposed to biocides such as benzalkonium chloride (Braoudaki and Hilton, 2004; Bore et al., 2007; Abdelaziz et al., 2019) and chlorhexidine (Braoudaki and Hilton, 2004; Wand et al., 2017; Verspecht et al., 2019) and reviewed elsewhere (Kampf, 2018). In the field, decreased susceptibility to biocides in association with antibiotic resistance was observed through the co-resistance phenomena (Cho et al., 2013; Gomaa et al., 2017). In this case, the genes allowing bacterial survival in both biocides and antibiotics are transmitted together due to the co-occurrence in the same genetic element such as a plasmid or integron (Chapman, 2003).

Further analysis of the mutant genomes resulting from ALE experiments or collected from the environment provides hints on the genetic basis of biocide adaptation. For example, mutations upregulating the efflux protein SmvA and in the two-component regulator PhoPQ in *Klebsiella pneumoniae* adapted to chlorhexidine were associated with resistance to this biocide (Wand et al., 2017). The membrane component TolC (Ferhat et al., 2009), the regulation of OmpF porin levels (Sakagami et al., 1989), and other mechanisms recently reviewed (Pereira and Tagkopoulos, 2019) were associated with resistance to benzalkonium chloride. Tolerance to hydrogen peroxide is mediated by a pool of enzymes such as peroxidases (Mishra and Imlay, 2012). Ethanol tolerance is well-known in the yeast *Saccharomyces cerevisiae*, arising mostly from changes in the membrane composition (Snoek et al., 2016).

Understanding the genetic basis for adaptation to different biocidal chemicals is of utmost importance to establish the connections between biocide usage and antibiotic resistance. We have previously studied the *E. coli* transcriptomic response after exposure for 30 min and 12 h to 10 widely used biocides, with applications ranging from hospital and food industry disinfection to over-the-counter household products (Pereira et al., 2020). Here, we conducted a long-term ALE study of *E. coli* replicate populations (∼500 generations) in the presence of the same ten biocides (Table 1). We assessed the relative fitness of the evolved cell lines through growth curves and competition assays and their cross-stress phenotypes with exposure to three clinically relevant antibiotics. Mutant genome-wide re-sequencing informed about the genetic basis of the acquired (cross) resistance. Exposure to specific biocides selected for cross-resistant and biofilm-forming bacteria, and genes previously associated with antibiotic resistance were mutated in the biocide-evolved strains.

**TABLE 1:** Biocides (antiseptics and disinfectants) utilized in this work.

Biocide	Abbreviation	Concentration (evolution)	Neutralizer (competition assay)	Group
Benzalkonium chloride	BENZ	4 mg/L	Lecithin 0.5% tween 80 1%	Cationic agent (QAC)
Chlorhexidine	XID	1.65 μM	Lecithin 0.5% tween 80 1%	Cationic biguanide
Chlorophene	PHE	0.5 mM	Lecithin 0.5% tween 80 1%	Halogenated phenolic
Glutaraldehyde	GLUT	31 μM	Sodium bisulfite 1%	Aldehyde
Hydrogen peroxide	H_2_O_2_	200 μM	Sodium thiosulfate 1%	Peroxygen
Ethanol	ETOH	4.25% (v/v)	Dilution only	Alcohol
Isopropanol	ISOP	2.5% (v/v)	Dilution only	Alcohol
Peracetic acid	PERA	90 μM	Sodium thiosulfate 1% tween 80 1%	Peroxygen
Povidone-iodine	POV	67 μg/mL	Sodium thiosulfate 1%	Halogen
Sodium hypochlorite	SOD	3.6 μM	Sodium thiosulfate 1%	Chlorine

*Concentrations of biocides to which *E. coli* cells were exposed during evolution experiments and neutralizers applied after competition assays.*

## Results

### Evolution in Biocides Result in Strains With Decreased Susceptibility to Both Biocides and Antibiotics

**FIGURE 1:**
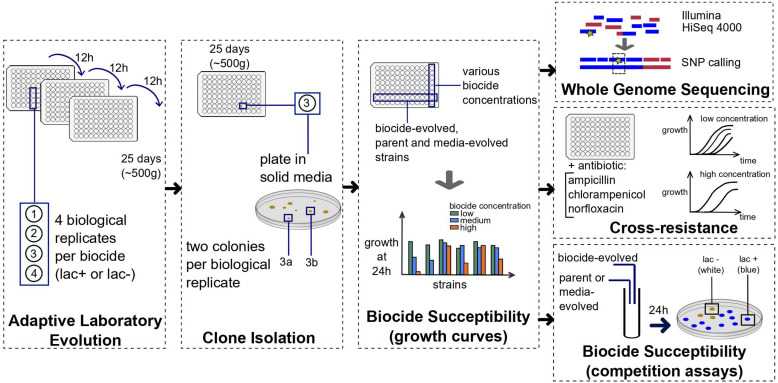
Summary of the methods used to obtain the evolved strains and the resistance and cross-resistance data. Four biological replicates were evolved in each of the ten biocides listed in Table 1 for approximately 500 generations. For each biological replicate, two colonies were picked (“a” and “b”), and each clone tested for biocide susceptibility with growth curves in 96 well-plates. Between two and four clones per biocide were selected based on growth curves and sequenced, tested for cross-resistance to antibiotics and biocide susceptibility with competition assays against the parent and media-evolved strains. Details are described in section “Materials and Methods.”

**FIGURE 2:**
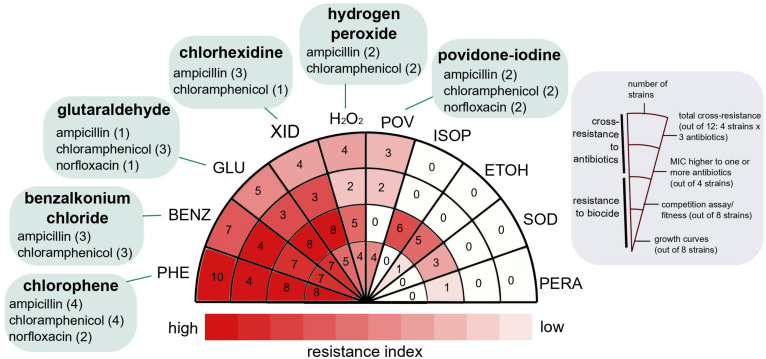
Resistance and cross-resistance distribution for strains evolved in ten widespread use biocides. The resistance index was calculated as described in section “Materials and Methods,” based on the resistance to the biocide (growth curves and competition assays) and cross-resistance to three medically relevant antibiotics. Biocides at the left side of the wheel (darker red) were more likely to select for strains with higher fitness potential and cross-resistant to antibiotics. The antibiotics to which the evolved strains were cross-resistant are indicated in green, and the number of strains exhibiting cross-resistance to the specific antibiotic is in parentheses. For details, see Table 2.

We evolved *E. coli* MG1655 in the presence of sub-inhibitory, constant concentrations of 10 ubiquitous biocides for 500 generations (Figure 1 and Supplementary Figure 1). There were striking differences between the evolved strains, in terms of susceptibility decrease to the biocides and the development of cross-resistance to antibiotics (Figure 2).

Adaptive laboratory evolution of *E. coli* in sub-inhibitory and constant concentration of biocides selected for strains resistant to higher concentrations than the ones applied for the evolution experiments. In five out of ten biocides (GLUTA, XID, PHE, BENZ, and H_2_O_2_), at least one evolved, biological replicate exhibited decreased susceptibility to concentrations higher than the one used for ALE as measured by two different methods: individual growth curves and competition assays with the parent and media-evolved strains (Figure 2 and Supplementary Figures 2, 3). For the remaining biocides (POV, ISOP, ETOH, SOD, and PERA), decreases in susceptibility were detected by a single method (Figure 2 and Supplementary Figures 2, 3).

Adaptive laboratory evolution of *E. coli* in the presence of biocides selected for strains cross-resistant to medically relevant antibiotics in 42.5% of the recovered strains (17 out of 40). Six out of ten biocides (GLUTA, XID, PHE, POV, BENZ, and H_2_O_2_) selected for strains cross-resistant to at least one of the following antibiotics: ampicillin, chloramphenicol, and norfloxacin (Figure 2 and Table 2). In addition to the strains from this work, we evaluated the cross-resistance to antibiotics of strains previously evolved by our group (Dragosits et al., 2013; Zorraquino et al., 2017) in other stresses such as osmotic, acidic, and butanol. From eight biological replicates tested, only one had MIC values mildly higher than the parent strain (Supplementary Table 1), suggesting that antibiotic cross-resistance selection is not conditional of overall cellular stress, but rather specific stress-causing chemicals.

**TABLE 2:** Strains evolved in biocides and cross-resistant to antibiotics.

Strain	Number of antibiotics (type) (MIC in μg/mL)	Gene or genomic region with mutation(s)
phe 3b	3 (amp, cm, nor) (16,32,0.25)	*ompR*
pov 1b/2a	3 (amp, cm, nor) (16,16,0.25)	*ompR*, intergene (upstream *pyrE*)
glu 2b	3 (amp, cm, nor) (16,8,0.25)	*pyrE*, *yeaW*
phe 2a	3 (amp, cm, nor) (8,16,0.125)	*envZ*
phe 4b	2 (amp, cm) (8,16)	*envZ*
xid 4b	2 (amp, cm) (8,8)	*mlaA*
phe 1b	2 (amp, cm) (4,16)	*acrR*, *rimP*
h_2_o_2_ 1a	2 (amp, cm) (4,8)	*rpoC*
h_2_o_2_ 3a	2 (amp, cm) (4,8)	*icd*, *rpoB*, *rpoC*, intergene (upstream *katG*)
benz 2b	2 (amp, cm) (4,8)	*gshA, rrfF*, intergene (upstream *mdfA*)
benz 3b	2 (amp, cm) (4,8)	Intergene (upstream *mdfA*)
benz 4b	1 (cm) (8)	Intergene (upstream *mdfA*)
glu 1a	1 (cm) (8)	*aes, yqhC*
glu 4a	1 (cm) (8)	*icd, rpoA, yqhC*
xid 2b	1 (amp) (4)	*pgpA*, intergene (upstream *isrC, flu*)
xid 3b	1 (amp) (4)	*rpoC*, *ymfE*, intergene (upstream *cdgI*)
benz 1b	1 (amp) (4)	*rpoB*

*amp, ampicillin; cm, chloramphenicol; nor, norfloxacin. The minimum inhibitory concentration (MIC) for the parent strain was 2 μg/mL in amp, 4 μg/mL in cm, and 0.031 μg/mL in nor. Cross-resistance was defined as a MIC at least 2 times higher than the parent strain. The genes or genomic regions in which a mutation was observed are indicated. For a complete list of alleles with the position and base changed compared with the parental allele, see Supplementary Table 2.*

Each of the cross-resistant strains from this work exhibited between one and three mutated genes, and seven cross-resistant strains had mutations in the upstream region of a gene (Table 2). On several occasions, the same gene (or genomic region, such as upstream a given gene) was mutated in different biological replicates, generating different alleles and evidencing the importance of such genes for survival in biocides (Supplementary Table 2). The mutations observed in cross-resistant strains and discussed next were distinct from those observed in media-only evolved strains and in the re-sequenced parent strain, which indicated that the presence of the biocide was the driver for the selection (Supplementary Table 2).

### Common Mutations Between Biocide and Antibiotic Evolved Strains Correlate With Cross-Stress Resistance

We investigated whether the overlap of mutations after evolution in biocides and antibiotics can predict antibiotic cross-resistance using data from MutationDB (Wang et al., 2018). Indeed, a higher overlap of mutations indicated a higher propensity of the strain to be less susceptible to the antibiotic (*p*-value = 0.002). At the same time, no correlation was observed for overlap with other groups (*p*-value = 0.772 for “no stress” and *p*-value = 0.5 for “other stress”) (Supplementary Figure 4). Although most of the non-cross-resistant strains had no or few matches to strains from MutationDB evolved in antibiotics, there was a striking exception for *pyrE* gene and intergenic region (Supplementary Figure 4A), which was mutated in five strains, three out each were non-cross-resistant (Supplementary Table 2). Mutations in this region appeared in both “no stress” and “antibiotic stress” categories of MutationDB (Wang et al., 2018; Supplementary Table 3).

### The Genetic Basis of Cross-Stress Resistance

**FIGURE 3:**
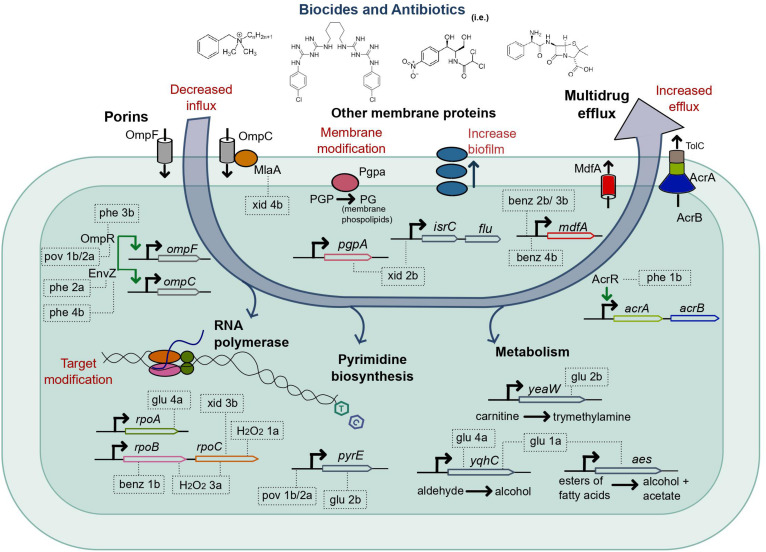
Mutations selected by the adaptive laboratory evolution of *E. coli* in biocides in strains cross-resistant to antibiotics. The systems and processes of the *E. coli* cell, which exhibited mutations after ALE in biocides, are shown in bold. The evolved strains cross-resistant to at least one antibiotic are represented inside dashed rectangles, which point to the gene or gene region with a detected mutation; for a complete list of mutations, see Table 2. The strains glu 2b, glu 1a, and xid 2b had only mutations not previously associated to antibiotic resistance (there is no precise mechanism reported in the literature), and the gene functions in the cell (Kanaya et al., 1998; Turnbough and Switzer, 2008; Lu et al., 2011; Koeth et al., 2014; Wang et al., 2015; Laganenka et al., 2016) are indicated for reference. Xid, chlorhexidine; pov, povidone-iodine; phe, chlorophene; benz, benzalkonium chloride; glu, glutaraldehyde.

For 14 out of 17 cross-resistant strains that we obtained, one or more of the mutated genes have been previously implicated in resistance to antibiotics. Such genes included porin regulators and associated proteins, regulators of multidrug efflux proteins, and RNA polymerase subunits (Figure 3).

We observed mutations related to porins, both in *ompR* (unique mutations in strains phe 3b and pov 1b/2a) and *envZ* (unique mutations in phe 2a and phe 4b). The EnvZ/OmpR is a two-component regulatory system widely distributed in bacteria and mainly well characterized in *E. coli*. It functions in osmoregulation and regulates the expression of the outer membrane porins OmpF and OmpC (Forst et al., 1989). Also, the strain xid 4b had a mutation in *mlaA* (Figure 3 and Supplementary Table 2), which encodes for an outer membrane protein that interacts with OmpC and OmpF (Chong et al., 2015; Atif et al., 2017). Both downregulation and absence of *ompF* and *ompC* have been associated with resistance to multiple antimicrobials (Harder et al., 1981; Yigit et al., 2002; Chetri et al., 2019). We verified that the expression of *ompF* and *ompC* was altered in all the strains mentioned above (phe 3b, pov 1b/2a, and phe 2a) compared to the parent strain when measured with qPCR (Supplementary Figure 5). The evolved strains which exhibited mutations related to this pathway (phe 2a, phe 3b, pov 1b/2a, and xid 4b) were less susceptible to the biocide in which they evolved (Supplementary Figures 2, 3) and cross-resistant to antibiotics (Table 2).

**FIGURE 4:**
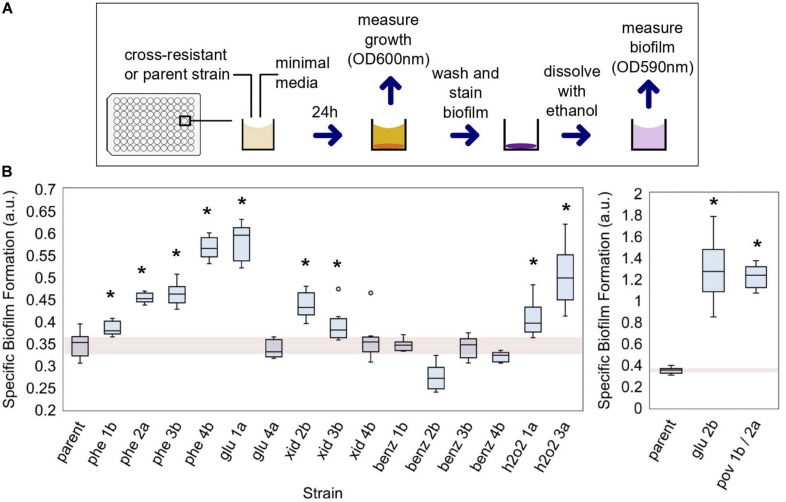
The capability of cross-resistant strains to form biofilms. **(A)** Biofilm was measured with a colorimetric method adapted from Christensen et al. (1985); see section “Materials and Methods” for details. **(B)** The evolved strains which were also cross-resistant to at least one antibiotic are shown and compared to the parent strain. The specific biofilm formation (SBF) was determined in minimal media. a.u., arbitrary units. A single asterisk indicates strains in which SBF was significantly different than that of the parent strain (*p*-value < 0.05; see Supplementary Table 5 for the complete list of *p*-values).

Exposure of *E. coli* to benzalkonium chloride (BENZ) selected for mutations upstream of the gene for the multidrug efflux protein MdfA in three out of four biological replicates (Supplementary Table 2 and Figure 4), with two unique mutations, in benz 2b/3b and benz 4b. No additional mutations were observed in benz 3b and benz 4b. Whether the mutation observed in both benz 2b and 3b strains was present in very low frequency in the parent strain (not captured in the re-sequencing; see Supplementary Table 2) or resulted from cross-contamination, is unclear. However, the selection for such mutation twice and for a different mutation in the same region in benz 4b, and the absence of additional mutations in two strains, evidences the clear importance of MdfA for survival in this biocide. In accordance, the multidrug efflux protein MdfA has been implicated in resistance to a wide range of chemicals (Edgar and Bibi, 1997). The MICs in benzalkonium chloride for the strains benz 2b, benz 3b, and benz 4b were higher than for the parent and M9-evolved controls (Supplementary Figure 2). The strains evolved in BENZ also exhibited higher fitness in competition assays (Supplementary Figure 3), except for the strain benz 2b against the parent strain.

The strain phe 1b, evolved in chlorophene, had mutations in both *acrR* and *rimP* (Supplementary Table 2) and exhibited decreased susceptibility to chlorophene (Supplementary Figures 2, 3) and cross-resistance to ampicillin and chloramphenicol (Table 2). The transcriptional regulator *acrR* is involved in the regulation of *acrAB* (Figure 3), which encodes for multidrug efflux proteins and other proteins associated with drug resistance (Ma et al., 1996). In accordance, the strain’s expression of the multidrug efflux protein *acrA* was upregulated compared to the parent strain (Supplementary Figure 5). We have previously demonstrated the importance of *acrA* presence for *E. coli*’s tolerance to chlorophene and benzalkonium chloride (Pereira et al., 2020).

Unique missense mutations in the RNA polymerase subunits *rpoA*, *rpoB*, *rpoC* were present in 8 out of 40 evolved strains (Supplementary Table 2), or 5 out of the 17 cross-resistant ones (Table 2 and Figure 3). Such mutations were selected for in strains exposed to BENZ (benz 1b), GLU (glu 4a), H_2_O_2_ (h_2_o_2_ 1a, h_2_o_2_ 3a), PERA (pera 4a), XID (xid 3a), and cells evolved in media only (M9-ev 2). Mutations in *rpoB* and *rpoC* are common in evolution experiments (Wang et al., 2018) and have been observed before (Zorraquino et al., 2017). They were previously associated with increased evolvability (Barrick et al., 2010) and resistance to rifampicin (Mariam et al., 2004), cephalosporin (Lee et al., 2013), and vancomycin (Matsuo et al., 2015).

### Genes for Enzymes and Membrane Maintenance Are Associated With Cross-Resistance to Antibiotics

Not all the mutated genes in the strains evolved in biocides and cross-resistant to antibiotics could be immediately related to antibiotic resistance mechanisms based on the current data available in the literature. From the cross-resistant strains with mutations in genes not previously associated with antibiotic resistance, the double mutant glu 2b was especially interesting. The strain glu 2b exhibited decreased susceptibility to the biocide in which it evolved (Supplementary Figures 2, 3) and cross-resistance to all the antibiotics assayed (Table 2). There were two single nucleotide polymorphisms (SNP) in the strain, in the genes *yeaW* and *pyre* (Figure 3 and Supplementary Table 2). There is no clear relationship between the genes (Supplementary Figure 6). *yeaW* encodes for part of a two-component oxygenase/reductase involved in the conversion of trimethylamine (Koeth et al., 2014; Wang et al., 2015). The *pyrE* gene encodes for an orotate phosphoribosyl transferase involved in the biosynthesis of pyrimidine nucleotides (Turnbough and Switzer, 2008). We validated the role of the observed mutations with single gene repair (restoring the wild-type allele). Although the gene repair for each gene in isolation did not fully restore the parent phenotype, both of the repaired strains showed increased sensitivity to the antibiotics. Average growth reductions in the presence of antibiotics compared to the non-repaired glu 2b amounted to 36 and 42% in chloramphenicol, 86 and 71% in ampicillin, and 8 and 7% in norfloxacin, for *yeaW* and *pyrE* gene repairs, respectively (Table 3).

**TABLE 3:** Percent reduction in biofilm formation and growth (as OD600 nm averages ± standard deviation) values for single gene repaired strains compared the respective evolved (non-repaired) strain.

Assay	Non-repaired strain/gene repaired	% Reduction
Biofilm	glu 1a/*aes*	45.3 ± 2.7
Biofilm	glu 2b/*yeaw*	37.9 ± 5.0
Biofilm	glu2b/*pyre*	22.1 ± 4.5
cm	glu 1a/*aes*	80.7 ± 5.4
cm	glu 2b/*yeaw*	35.9 ± 5.3
cm	glu2b/*pyre*	42.2 ± 3.9
amp × 12 h	glu 2b/*yeaw*	86.3 ± 1.9
amp × 12h	glu2b/*pyre*	70.5 ± 4.3
nor	glu 2b/*yeaw*	8.0 ± 0.2
nor	glu2b/*pyre*	7.2 ± 0.7

*Antibiotic susceptibility was measured at 24 h and a concentration two times lower than the MIC value of the indicated evolved strain, except for ampicillin. cm, chloramphenicol; amp, ampicillin; nor, norfloxacin.*

The strain glu 1a, evolved in glutaraldehyde, had mutations in both *yqhC* and *aes* (Supplementary Table 2). The strain was less susceptible to glutaraldehyde (Supplementary Figures 2, 3) and cross-resistant to chloramphenicol (Table 2). Since not all the strains harboring mutations in *yqhC* exhibited a cross-resistant phenotype, we hypothesized that the effect arose from the mutation in *aes* or a synergic effect between the genes. The gene *aes* encodes for an acetyl esterase involved in the hydrolysis of p-nitrophenyl esters of fatty acids (Kanaya et al., 1998; Figure 3). In accordance, the single gene repair for *aes* partially restored the sensitive phenotype of the parent strain, resulting in 80% less growth than the glu 1a strain in the presence of chloramphenicol (Table 3).

Finally, the strain xid 2b, evolved in chlorhexidine, had mutations in *pgpA* and upstream of *isrC*/*flu* (Supplementary Table 2 and Figure 3). The strain had impaired growth and was moderately less susceptible to chlorhexidine (Supplementary Figures 2, 3) and ampicillin (Table 2). The phosphatidylglycerophosphatase encoded by *pgpA* participates in the catalytic processes of phospholipids of the inner and outer membrane in *E. coli* (Lu et al., 2011) while the self-recognizing antigen 43 (Ag43) autotransporter encoded by *flu* plays a role in protection against hydrogen peroxide and biofilm formation (Laganenka et al., 2016). In accordance, the strain xid 2b exhibited a higher capacity for biofilm formation than the parent strain (Figure 4).

### Strains Cross-Resistant to Antibiotics Are Biofilm-Forming

The formation of bacterial biofilms has implications for disinfection and human disease (Abreu et al., 2013; Nobile and Johnson, 2015). Bacteria can better survive stressful conditions when organized in biofilms (Mah and O’Toole, 2001; Bridier et al., 2011). Given that, we assessed the biofilm formation capability of the strains which exhibited cross-resistance to antibiotics (Figure 4A). ALE of *E. coli* in the presence of biocides selected for strains more capable of forming biofilms (Figure 4B). In 11 out of 17 isolates, the specific biofilm formation (SBF) was superior to the parent strain (*p*-value < 0.05), when measured without the presence of biocides (media-only). The cross-resistant strains evolved in benzalkonium chloride did not improve their biofilm-forming capabilities. For glu 2b and pov 1b/2a strains, the SBF was significantly superior to the other strains, and more than three times that of the parent (1.26 and 1.22, respectively) (Figure 4 and Supplementary Table 4).

*Escherichia coli* MG1655 (our parent strain) has naturally a poor biofilm-forming capacity (Ghigo, 2001; Reisner et al., 2003) and we classified it as non-adherent based on Christensen’s method for classification (Christensen et al., 1985). Exposure to biocides changed this phenotype, and six cross-resistant strains (phe2a, phe3b, phe4b, glu1a, glu2b, and pov1b/2a) were classified as adherent based on the parameters of the method.

We performed gene repairs on glu2b and glu1a strains. The repaired strains partially restored the parental phenotype. The SBF for the strains with gene mutation repairs was reduced by 37 and 22% for *yeaW* and *pyrE*, respectively, compared to the non-repaired strain glu 2b (Table 3), suggesting a contribution of both mutations for biofilm formation. The single gene repair for *aes* in the glu 1a strain also resulted in a reduction in the SBF compared to the non-repaired strain, by 45% (Table 3).

## Discussion

In this work, we focused on the evolutionary potential and the genetic basis of bacterial adaptation to biocides and their cross-resistance to antibiotics. Our results suggest that biocides invoke disparate evolutionary trajectories, with some promoting the emergence of cross-resistance, while others do not. Chlorophene, benzalkonium chloride, chlorhexidine, and glutaraldehyde were the top biocides in the first category. As such, the intelligent control of their use is appropriate when the goal is to decelerate the emergence of antibiotic resistance. Both benzalkonium chloride and chlorhexidine have been previously flagged by their potential to promote cross-resistance, although the genetic basis was not investigated (Kampf, 2018). In contrast to existing literature (Kampf, 2018), we report on the cross-resistance potential for exposure to glutaraldehyde.

From the biocides tested, evolution in povidone-iodine exhibited unique dynamics, with population collapse in most biological replicates after a few generations, for otherwise similar conditions (growth in constant, sub-inhibitory concentration of biocide equivalent to 50% growth inhibition in 12 h; see sections “Materials and Methods” and “Supplementary Methods”). Despite claims that resistance does not emerge in this biocide (Reimer et al., 2002; Lachapelle et al., 2013; Kampf, 2018) we isolated clones with increased capacity to survive in the biocide compared to non-evolved, which were also less susceptible to antibiotics. Such occurrence suggests that resistance selection from povidone-iodine exposure is a rare but potentially possible event and may explain contradictory results to the literature.

Out of six biocides that selected for cross-resistant strains, four included strains with mutations in proteins of the membrane and their regulators, including those involved in the transport of chemicals such as antibiotics in and out of the cell (Figure 3). Membrane-related mutations were overrepresented amongst cross-resistant strains compared to non-cross-resistant (Supplementary Table 2), which suggests that the potential of a given biocide to select for such mutations is associated with a higher risk for its use.

Most of our biocide-evolved, cross-resistant strains were also better biofilm-forming than the non-evolved parent, which has potential health implications. The ability of bacteria to form biofilms reduces the antimicrobial effectiveness (Costerton et al., 1999; Abreu et al., 2013). Two of the evolved strains had a considerable increase in biofilm formation: pov 1b/2a and glu 2b. For pov 1b/2a, such effect can be attributed to a mutation in *envZ* given the known role of the gene for the regulation of flagellar and curli genes (Brombacher et al., 2003; Prüß, 2017). However, the biofilm increase in glu 2b could not be easily explained based on the current literature and suggests a connection between the genes affected (*yeaW* and *pyrE*) and the phenotype observed. The repair of each gene mutation individually partially restored the non-biofilm forming phenotype, suggesting a synergic relationship between the gene products. Further experiments would be needed to clarify such a link.

Here, to simulate conditions of residual environmental concentrations following biocide usage, we opted for maintaining the biocide concentration constant during ALE, while other researchers (Braoudaki and Hilton, 2004; Bore et al., 2007; Lázár et al., 2014; Oz et al., 2014; Wand et al., 2017) have selected for highly resistant strains by exposure to stepwise increasing concentrations. As higher selection pressures result in the selection of more cross-resistant strains than milder conditions (Oz et al., 2014), we expect that more resistant strains, or even strains with collateral sensitivity, will emerge in such settings (Maltas et al., 2020), if the goal is that of strain engineering.

We limited our study to a single bacterial species in a laboratory environment to better compare biocides and control test conditions. In the field, however, other mechanisms for resistance emergency and maintenance in bacterial populations may be at play. For example, the mechanism of horizontal transfer of genetic elements, not approached by this work, is an important driver of resistance of bacterial populations in the field (Lerminiaux and Cameron, 2019). Tolerance to quaternary ammonium compounds and chlorhexidine in clinical and environmental isolates has been associated to antibiotic resistance through the co-occurrence of resistance genes in integrons or plasmids (Chapman, 2003; Cho et al., 2013; Gomaa et al., 2017).

Biocides are of fundamental importance to control and eliminate pathogens in high-risk settings such as hospitals. Our work highlights the need to stimulate the responsible use and discard of such products to avoid unnecessary bacterial exposure and potential selection pressure for cross-resistant strains. In addition, we acknowledge that further analysis of other bacterial species and clinical isolates of *E. coli*, as well as mixed-species communities, to multiple biocides in a similar fashion of this work have the potential to amplify the knowledge provided here.

## Materials and Methods

### Biocides

Benzalkonium chloride (MP Biomedicals), hydrogen peroxide (Macron), peracetic acid (Sigma-Aldrich), sodium hypochlorite (Sigma-Aldrich), glutaraldehyde (Amresco), chlorhexidine (Aldrich), chlorhexidine gluconate (Spectrum), and povidone-iodine (Sigma) stock solutions were prepared by dilution in sterile, demineralized water. Chlorophene (Aldrich) stock solution was prepared by dilution in ethanol (Sigma-Aldrich). All solutions, including ethanol (Sigma-Aldrich) and isopropanol (Spectrum), were sterilized with 0.22 μm filters and kept at 4°C. Working solutions were prepared daily by further dilutions in sterile, demineralized water.

### Antibiotics

Three representative antibiotics with various modes of action in the cell and medically relevant for the species utilized in this work were selected for cross-resistance evaluation. The antibiotics selected were: ampicillin (Roche), chloramphenicol (Calbiochem), and norfloxacin (Sigma-Aldrich). Ampicillin acts in the cell membrane, chloramphenicol affects protein synthesis (ribosomal action), and norfloxacin blocks DNA replication (DNA gyrase). Stock solutions (20 mg/mL) were prepared by dilution in sterile demineralized water, ethanol, and 0.1M HCl, respectively, and stored at −20°C. Working solutions were prepared as needed by dilution in sterile demineralized water.

### Bacterial Strains

*Escherichia coli* MG1655 was used for ALE experiments. A strain with a SNP in *lacY* in position 363155 (Δlac) was used as a started strain in ALE experiments indexed 3 or 4 (i.e., glu 3a). The strains were kept at −80°C with 15% glycerol. *E. coli* BW25113 knockout strains from the Keio Collection were used when indicated (Baba et al., 2006). Additional MG1655 evolved strains tested were obtained previously (Dragosits et al., 2013; Zorraquino et al., 2017).

### Adaptive Laboratory Evolution Setup

For the evolution experiments, four biological replicates for each of the 10 biocides (exception: povidone-iodine) tested were evolved for approximately 500 generations (approximately 10 generations per 12 h period). Part of the replicates were derived from *E. coli* MG1655 wild-type (parent +) and part from *E. coli* MG1655 Δlac (parent −) genotype used previously by our group (Dragosits et al., 2013; Zorraquino et al., 2017). The evolution was performed at 37°C with agitation (Synergy HT, BioTek) in 96-well, flat bottom, polystyrene non-treated plates (Costar, Corning) containing 200 μL of Minimal media with 0.4% glucose (M9 glucose) and one of the biocides. The biocide concentrations used for evolution are listed in Table 1. The first inoculum consisted of 2 μL of cells grown for 12 h in M9 glucose. Every 12 h, approximately 2 μL of cells (adjusted to a constant concentration of 2 × 10^5^ – 3 × 10^5^ CFU/μL) were transferred to new wells containing fresh media and the biocide, so that cells would remain at exponential (log) phase for most of the duration of the experiment. The volume of cells added at each transfer was adjusted based on the OD600 nm to keep the initial concentration of cells after each transfer approximately constant. After growth, glycerol was added to the wells to a final concentration of 15% (v/v), and the plates were stored at −80°C. Next, 2–3 μL of evolved cells were streaked into LB agar plates and grown overnight at 37°C. Both *E. coli* MG1655 wild-type and *E. coli* MG1655 Δlac were evolved in M9 glucose media without the addition of any biocide for 500 generations for comparison (M9-ev + and M9-ev −). Two colonies were randomly picked from each plate, grown overnight in M9 glucose media, and stored with 15% glycerol (v/v) at −80°C.

### Growth Curves for Biocide Susceptibility

Each one of the 82 evolved clones (500 generations) were tested for biocide susceptibility in 96 well-plates with 200 μL M9 glucose media containing five concentrations of the biocide in which they were evolved. The parent strains (not evolved) and the M9-evolved strains (evolved in media only, without biocide) were tested in parallel for comparison. The assays were performed at least in duplicate, and the error was calculated as the standard deviation divided by the square root of the number of replicates.

### Competition Assays for Biocide Susceptibility

Four evolved clones from biological replicates in each biocide (composing a total of 40 independently evolved clones) were selected based on the growth curves and tested for biocide susceptibility using competition assays (Dragosits et al., 2013; Zorraquino et al., 2017). For this assay, cells were grown overnight in 2 mL of M9 glucose, and the OD600 nm was adjusted to 1.0. For a given assay, 100 μL of an evolved clone and 100 μL of a control (either the parent strain or the strain evolved in M9 without biocide), were mixed in a tube containing 10 mL of M9 glucose and a biocide. The volume was split into three tubes, and a sample was taken from one of the tubes, neutralized, diluted in saline, and plated in X-Gal IPTG LB agar to determine the cell concentration at time zero. The cells competed for 24 h, and a sample of each of the remaining two tubes was taken, neutralized with an appropriate solution (Table 1), diluted in saline and plated in X-Gal IPTG LB agar (0.25 mM IPTG Isopropyl b-D-1-thiogalactopyranoside and 40 mg/ml X-gal bromo-chloro-indolyl-galactopyranoside). The agar plates were incubated at 37°C and the cell count (CFU) was determined. Dilutions were determined previously to result in an average of 100 CFU per plate. To differentiate between the colonies for the evolved clone being tested and the control, one of which had the genotype Δlac (white colonies), and the other did not (blue colonies). A minimum of two concentrations of each biocide were tested. The selection rate was calculated as a measure of fitness (see section “Supplementary Methods” for details). The error was calculated as the standard deviation divided by the square root of the number of replicates.

### Cross-Resistance to Antibiotics

Selected *E. coli* clones were evaluated for cross-resistance to antibiotics with growth curves in 96-well plates. The inoculum used in the plates was prepared from cultures grown from −80°C freezer stocks overnight (14–18 h) in M9-glucose media. The standards for the broth microdilution method from the Clinical and Laboratory Standards Institute (2012) were followed, with few adaptations. In brief, in each well, 2 μL of diluted overnight cell cultures were mixed with 100 μL of M9-glucose media containing the antibiotic. The overnight cell stocks were diluted to a fixed concentration (based on OD600 nm) to result in 2 to 8 × 10^5^ CFU per well of the final plate. Plates were incubated at 37°C with agitation in a plate reader (Synergy HT, BioTek) for up to 24 h and the OD600 nm was captured every 15 min.

### Genome Sequencing

Four evolved clones (500 generations) from biological replicates in each biocide, and two replicates for povidone-iodine (composing a total of 38 clones) were selected based on the growth curves and competition assays for DNA-sequencing. In addition, two biological replicates of the cells evolved in M9 media only, and the parent strain (non-evolved) were also sequenced. Genome DNA was extracted according to the protocol for Gram-negative bacteria from Wizard Genomic DNA purification kit (Promega). The DNA was fragmented using Covaris E220 (microtube AFA fiber snap-cap for 130 μL, peak incident power 140 w, duty factor 10%, 200 cycles per bust, treatment time 70 s). Samples were stored at −20°C until the libraries were prepared according to the instructions of the KAPA LTP Library preparation Kit for Illumina Platforms Kit (KAPA Biosystems). The DNA concentration was determined with Qbit and/or Agilent Bioanalyzer 2100. Final pooled libraries were sequenced with HiSeq 4000 PE150 at the DNA Technologies and Expression Analysis Cores (Genome Center, University of California Davis).

### SNP and Short Indel Calling and Mutation Annotation

The reference genome sequence was NCBI| Reference Sequence U00096.3. For each sample, the reads were aligned to the *E. coli* K12 (strain MG1655) genome using the short-read alignment tool, Bowtie2 (version:2.3.5.1) (Langmead and Salzberg, 2012). The SNP and short indel mutations were called using an open-source tool, VarScan (Koboldt et al., 2009). The criterion for filtering variants is that the frequency of a variant is larger than 49%, and the *p*-value is less than 0.01. Note that the detected variants did not change if we used a higher *p*-value as a cut-off, whereas varied when we changed the cut-off for the frequency of a variant. The detected mutations were annotated to by synonymous, non-synonymous, or non-coding based on the coordinates and genetic code of protein-coding genes of *E. coli* using a custom script.

### qPCR

Samples were prepared by mixing the culture with a half volume of cold 5% phenol/ethanol (v/v), following by centrifugation for 10 min at 4000 rpm and 4°C. The supernatant was discarded, and the cells stored at −80°C. The RNA was extracted with the RNeasy mini kit (Qiagen) and RNAse-free DNAse set (Qiagen). The cDNA was prepared using a revert-aid first-strand cDNA synthesis kit (Thermo Fisher Scientific). The qPCR reaction was prepared using the powerup SYBR green master mix (Applied Biosystems). Plates were sealed with absolute qPCR seal (Thermo Fisher Scientific), spin down, and run using Viaa7 (Applied Biosystems). Results were analyzed using Quantstudio v1.3.

### Biofilm Assay

Biofilm was measured based on the crystal violet staining method adapted from Christensen et al. (1985). *E. coli* cells were grown overnight in M9 (minimal media), and 2 μL with OD600 adjusted to 0.1 were added to COSTAR 3997 tissue culture treated (Corning) 96 well plates containing 198 μL of M9. The plates were closed with a tight lid and incubated at 37°C for 24 h. The OD600 nm was measured using a plate reader (Synergy HTX, BioTek). The plate was washed twice with demineralized water, and the plate was let dry for 5 min. Next, 200 μL of 0.2% crystal violet was added to each well and incubated at room temperature for 5 min. The plate was washed four times with demineralized water and incubated at room temperature for 5 min. Next, 200 μL of ethanol was added to each well, the plate was mixed in the plate reader for 1 min, and the OD590 nm was measured.

The SBF was calculated by dividing the OD590 nm of the stained bacteria by the OD600 nm of bacterial growth after 24 h in minimal media. We used the *p*-values < 0.05 to determine whether the difference between the evolved-strain’s SBF was significantly different from the parent’s. *p*-Values were calculated with a one-tailed *t*-test for independent means for a minimum of eight replicates. We also used the classification from Christensen et al. (1985) to separate strains between non-adherent and adherent. In short, when the average OD590 nm for stained cells was higher than the average plus three standard variations of the control wells (wells without cells), the strain was considered adherent.

### Resistance Index

The resistance index for a given biocide (Figure 2) was calculated by the sums of (1) the total number of biocide-evolved strains which showed decreased susceptibility to the biocide compared to the parent strain in growth curve assays; which meant that the biocide-evolved strain grew better than the parent after 24 h of incubation in a given biocide concentration or was able to grow at a concentration higher than that of the parent (marked in red in Supplementary Figure 2), (2) the total number of competition assays in which the biocide-evolved strains were better fit (selection rate higher than zero) than the parent strain or the media-evolved strain (Supplementary Figure 3), (3) the number of biocide-evolved strains which were cross-resistant to at least one antibiotic (Table 2), (4) the total number of antibiotic-strain cross resistance combinations; i.e., four for H_2_O_2_: two strains evolved in H_2_O_2_ were cross-resistant to two antibiotics each (Table 2).

### Mutation Repair

We performed single-gene repairs on cross-resistant strains in which gene mutations have not yet been clearly associated with the observed cross-resistant phenotype. Mutations were repaired by replacing the gene and surrounding area (upstream or downstream) with the wild-type version obtained from the Keio Collection (Baba et al., 2006). Keio strains were selected to contain the kanamycin cassette in the neighborhood region of the gene being repaired, allowing for selection of repaired strains. The pKD46 (Adgene) recombination system, which contains the lambda red recombinase and a temperature-sensitive origin of replication, was used. The genomic region containing the kanamycin cassette and the gene to be replaced was amplified and transformed by electroporation into the evolved strain containing the pKD46 plasmid. The detailed method is in Supplementary Material (SOM) and Supplementary Figure 7.

### MutationDB Data Extraction and Analysis

We have previously created a database (MutationDB) containing *E. coli*’s mutations after ALE in various media and chemicals (Wang et al., 2018). Here, we interrogated whether the knowledge in the database would be informative to indicate which evolved strains from this study would have a phenotype of cross-resistance to antibiotics. We captured data from MutationDB for the conditions in which mutations were observed in the same gene (or intergenic region) as the strains evolved in biocides (Supplementary Table 3). We separated the MutationDB conditions into three groups: “antibiotic stress,” “other stress,” and “no stress,” and our mutated strains in two groups: without (*n* = 17) (Supplementary Figure 4A) and with (*n* = 18) (Supplementary Figure 4B) cross-resistance to at least one antibiotic.

## Data Availability Statement

The datasets presented in this study can be found in online repositories. The names of the repository/repositories and accession number(s) can be found below: NCBI BioProject accession number: PRJNA694447, https://www.ncbi.nlm.nih.gov/bioproject/PRJNA694447.

## Author Contributions

IT and BM conceived the project, designed the experiments, and revised the manuscript. BM performed the experiments. XW analyzed the genomic data. BM, IT, and XW wrote the manuscript. All authors contributed to the article and approved the submitted version.

## Conflict of Interest

The authors declare that the research was conducted in the absence of any commercial or financial relationships that could be construed as a potential conflict of interest.
