# Supplementary material for: Biocide-Induced Emergence of Antibiotic Resistance in Escherichia coli
Source: Front Microbiol. 2021 Feb 26;12:640923. doi: 10.3389/fmicb.2021.640923 (PMC7952520; doi:10.3389/fmicb.2021.640923)
Supplement: Supplementary file 1 [file Data_Sheet_1.zip › Supplementary Methods and Table 1.docx]

**Biocide-induced emergence of antibiotic resistance in *Escherichia coli***

Beatriz Merchel Piovesan Pereira^1,2^, Xiaokang Wang^2,3^, Ilias Tagkopoulos*^2,4^

1. Microbiology Graduate Group, University of California, Davis, USA
2. Genome Center, University of California, Davis, CA, USA
3. Biomedical Engineering Graduate Group, University of California, Davis, USA
4. Department of Computer Science, University of California, Davis, USA

*Corresponding author

**SUPPLEMENTARY INFORMATION**

**Supplementary methods**

**Selection rate (fitness) calculation in competition assays.** Selection rates (r) were used instead of relative fitness (W) as a comparative measure for two strains in competition assays. We have used fitness (W) previously (Dragosits et al. 2013; Zorraquino et al. 2017), however, exposure to antimicrobials frequently leads to decline in abundance (killing) instead of growth. Additionally, one evolved population can be significantly more fit than the other. In both cases, the use of selection rates (r) are recommended (Travisano and Lenski 1996; Lenski, n.d.).

The selection rate is the difference between the realized Malthusian parameter for strains A and B (mA and mB), which is calculated as follows:

$$r=mA-mB$$

$$r=\ln\left( \frac{A1}{A0} \right)-\ln\left( \frac{B1}{B0} \right)$$

In which A(i) and B(j) are the cell concentrations for strains A and B, at the beginning of the competition assay, or at 0 hours, (i=0, j=0) and at the end, or at 1 day, 24h (i=1, j=1).

For **Fig. S3**, the selection rates were calculated in terms of the biocide-evolved strain (A) compared to the parent or media-evolved (B). Selection rates higher than zero indicated an advantage of the biocide-evolved strain over the parent or media-evolved.

**Povidone-iodine sample selection.** For all biocides except povidone-iodine, four biological replicates (unique wells in 96-well plates) were evolved independently. The strain pov 2a originated from the pov 1b, hence, they were treated as replicates in most analysis. The isolates were collected from populations which evolved independently only for the final ~200 generations (they were split from each other at that moment of evolution). Multiple attempts were made to recover more biological replicates for povidone-iodine exposed populations, unsuccessfully. Even though the concentration of the biocide was not increased in between transfers, frequent population collapse events at subsequent inoculations were observed for such biocide.

**Gene repair.** The alleles (mutated genes) in the biocide-evolved strains were reverted to the wild-type alleles by recombination with a linear DNA molecule containing the wild-type gene allele and a kanamycin resistance cassette, using the lambda red system as described next. Biocide-evolved strains were made chemically competent and received the pkd46 plasmid (<https://www.ncbi.nlm.nih.gov/nuccore/AY048746>) by heat shock transformation. The plasmid contains a temperature sensitive origin of replication and the lambda red recombination system under araBAD promoter control. Cells were grown overnight at 30ºC with 50-100 µg/mL ampicillin, and next diluted 1:100 in 50 mL of fresh LB ampicillin with 10 mM L-arabinose for the expression of the lambda system. After three hours, cells were washed three times with 50, 20, and 5 mL of 10% ice-cold glycerol. The final pellet was resuspended in 1 mL and split into 1.5 mL tubes containing 40 µL of glycerol-washed cells each. The linear DNA was obtained from amplification of the desired region from the gDNA of Keio Collection strains. Keio strains that contained the kanamycin cassette next to the gene of interest (to be repaired) were used: ∆*yeaX* (JW1792) for *yeaW* repair, ∆*rph* (JW3618) for *pyrE*, and ∆*gsk* (JW0466) for *aes*. Primers were designed to amplify the kanamycin cassette and the wild-type allele of the gene of interest next to it as a single DNA strand, with overhangs for the recombination (**Fig. S7**). The linear DNA (1-4 µL) was transformed by electroporation and after the addition of 1mL of LB cells were recovered for 2-4h at 37ºC, pelleted by centrifugation, resuspended in 50-100 µL of 10% glycerol or LB, platted in LB kanamycin and incubated in 37-42ºC.

**Supplementary References**

Dragosits, Martin, Vadim Mozhayskiy, Semarhy Quinones-Soto, Jiyeon Park, and Ilias Tagkopoulos. 2013. “Evolutionary Potential, Cross-Stress Behavior and the Genetic Basis of Acquired Stress Resistance in Escherichia Coli.” *Molecular Systems Biology* 9 (1).

Lenski, Richard. n.d. “Comment On The Use Of Selection Rate Versus Relative Fitness.” http://myxo.css.msu.edu/ecoli/srvsrf.html.

Szklarczyk, Damian, Annika L Gable, David Lyon, Alexander Junge, Stefan Wyder, Jaime Huerta-Cepas, Milan Simonovic, et al. 2019. “STRING V11: Protein–Protein Association Networks with Increased Coverage, Supporting Functional Discovery in Genome-Wide Experimental Datasets.” *Nucleic Acids Research* 47 (D1): D607–D613.

Travisano, Michael, and Richard E Lenski. 1996. “Long-Term Experimental Evolution in Escherichia Coli. IV. Targets of Selection and the Specificity of Adaptation.” *Genetics* 143 (1): 15–26.

Zorraquino, Violeta, Minseung Kim, Navneet Rai, and Ilias Tagkopoulos. 2017. “The Genetic and Transcriptional Basis of Short and Long Term Adaptation across Multiple Stresses in Escherichia Coli.” *Molecular Biology and Evolution* 34 (3): 707–717.

**Supplementary Figures Legends**

**Figure S1**. Growth curves for independent biological replicates for the first 12 hours of evolution (0-12h). Biocide concentration used for evolution experiments was selected to cause growth delay (extended lag phase and/or reduced growth rate) compared to cells not exposed to any biocide in the first 12h hours of exposure. Growth was measured as OD600nm. M9-ev: cells not exposed to any biocide, evolved in media only; benz: benzalkonium chloride; etoh: ethanol; isop: isopropanol; glu: glutaraldehyde; pera: peracetic acid; xid: chlorhexidine; h2o2: hydrogen peroxide; sod: sodium hypochlorite; phe: chlorophene; pov: povidone-iodine.

**Figure S2**. Susceptibility of evolved strains to the biocide in which they evolved. Each graph shows the growth at 24 hours of the parent strains (parent + and parent -), the strains evolved in media only (M9-ev+ and M9-ev -) and the strains evolved in biocide at several biocide concentrations (indicated in the legends) in the minimal growth media. Biocide-evolved strains which showed decreased susceptibility to the biocide in which they evolved compared to the parent strain are shown in red. These were considered for the resistance index in **Fig 2**. The media contained one of the following: benz: benzalkonium chloride; etoh: ethanol; isop: isopropanol; glu: glutaraldehyde; pera: peracetic acid; xid: chlorhexidine; h2o2: hydrogen peroxide; sod: sodium hypochlorite; phe: chlorophene; pov: povidone-iodine.

**Figure S3**. Selection rate (fitness) of biocide-evolved strains compared to the parent or media-evolved strains, in the biocide in which they evolved. A selection rate higher than zero indicates that the corresponding biocide-evolved strain was more fit than the parent (orange) or media-evolved (blue) strain when growing in the presence of the biocide during the 24h assay. The concentrations used for each assay were as follows: benz: benzalkonium chloride (6.4 mg/L); etoh: ethanol (4%); isop: isopropanol (3.5%); glu: glutaraldehyde (50 µM); pera: peracetic acid (30 µM); xid: chlorhexidine (3.3 µM); h2o2: hydrogen peroxide (600 µM); sod: sodium hypochlorite (18 µM); phe: chlorophene (0.25 mM); pov: povidone-iodine (25 µg/mL).

**Figure S4**. Correlation between MutationDB data and evolved strains’ mutations**.** **A**. Smoothed histogram for the distribution of shared mutations in genes and intergenic regions between non-cross-resistant strains from this work and strains in MutationDB. Strains in MutationDB were divided in three groups based on the stress used during evolution: “no stress” (pink), “other stress” (blue) and “antibiotic stress” (green) B. Same as (A) for cross-resistant strains.

**Figure S5.** Expression of porin (A, B) and multidrug efflux (C) genes measured with qPCR **A**. The evolved strains which exhibited mutations in related pathways were compared to the parent strain (log2 fold-change), with and without the presence of biocide in the growth media preceding RNA extraction. The biocide tested was the same in which the cells evolved (i.e. phe for “phe 2a” strain), in the concentrations indicated in Table 1. **B**. log2 fold-change between the parent strain grown in the presence of the indicated biocide compared the parent strain grown in the absence of biocide. The biocides used were as follows: phe: chlorophene, pov: povidone-iodine, xid: chlorhexidine. **C.** Expression of the multidrug efflux *acrA* and *acrB*, regulated by *acrR*, in the evolved strain phe 1b compared to the non-evolved parent strain.

**Figure S6**. The highly cross-resistant strain glu 2b had mutations in unrelated genes which may play a role in novel resistance mechanisms. The gene network for *yeaw* (red) and *pyre* (green) built with STRING v.11 (Szklarczyk et al. 2019) shows no known direct connections between the genes when the number of interactors is set to <50 genes (The genes grouped separately from each other).

**Figure S7**. Design for the recovery of DNA and recombination for the repair of mutated genes from ALE experiments. An example is shown for *yeaW* repair (1). gDNA was extracted from JW1792 strain (containing the *kan* cassette in place of yeaX) and the region was amplified with primers designed to result in a minimum of 40 bp of homology (2) in each side of the DNA fragment used for electroporation (3). Recombination replaced the mutated gene (4) and inserted the *kan* resistance cassette for selection (5).

**Supplementary tables**

**Table S1**

| **Stress condition** | **ampicillin** | **chloramphenicol** | **norfloxacin** |
| --- | --- | --- | --- |
| Salt (NaCl) rep.1 | same | same | same |
| Salt (NaCl) rep.2 | 2x higher | same | 2x higher |
| Hydrogen peroxide rep 1 | same | same | same |
| Hydrogen peroxide rep 2 | same | same | same |
| Butanol rep 1 | same | same | same |
| Butanol rep 2 | 2x lower | 2x lower | same |
| Acidic rep 1 | same | same | same |
| Acidic rep 2 | 2x lower | same | same |

**Table S1.** MIC profiling in antibiotics for strains evolved in various stress conditions relative to the parent strain. Two biological replicates per condition are depicted.

**Table S2** (excel file). Complete list of mutations for ALE (adaptive laboratory evolved) strains.

**Table S3** (excel file). Complete list of shared mutations between evolved strains and strains from MutationDB used to populate the histograms in **Fig. 3**.

**Table S4.** (excel file). Specific biofilm formation (SBF) values of replicates used to calculate the biofilm specific formation average and corresponding p-values calculated using a one-tailed t-test for two independent means for the comparison with the control (parent strain).
