# Supplementary figures and images for: Biocide-Induced Emergence of Antibiotic Resistance in Escherichia coli
Source: Front Microbiol. 2021 Feb 26;12:640923. doi: 10.3389/fmicb.2021.640923 (PMC7952520; doi:10.3389/fmicb.2021.640923)

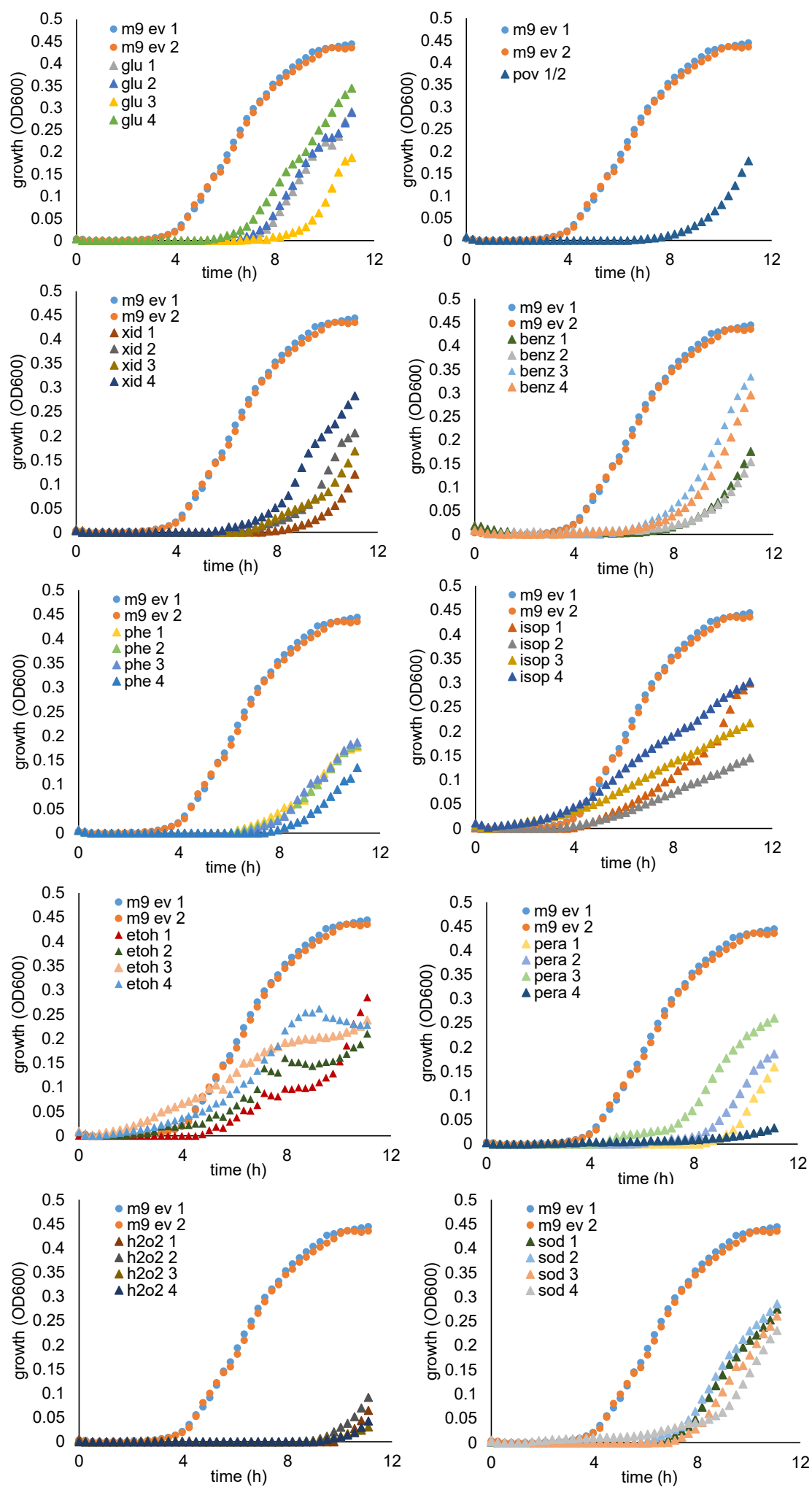

Supplement: Supplementary file 1 [file Data_Sheet_1.zip › Supplementary Figure 1.pdf]

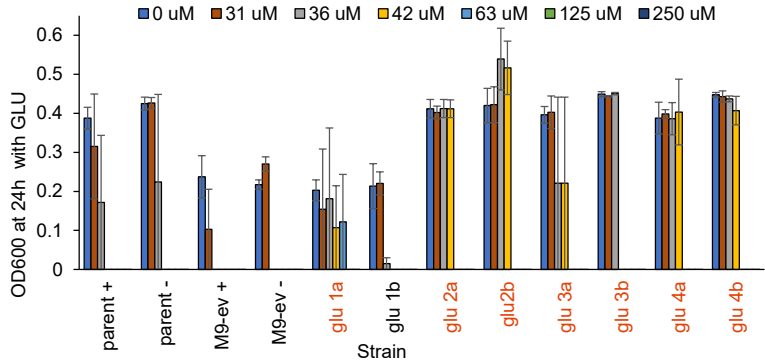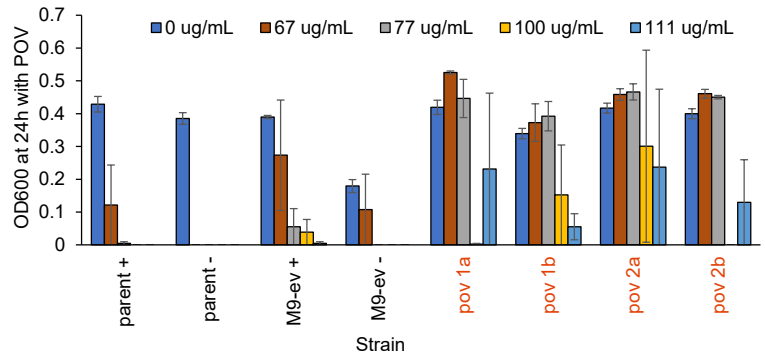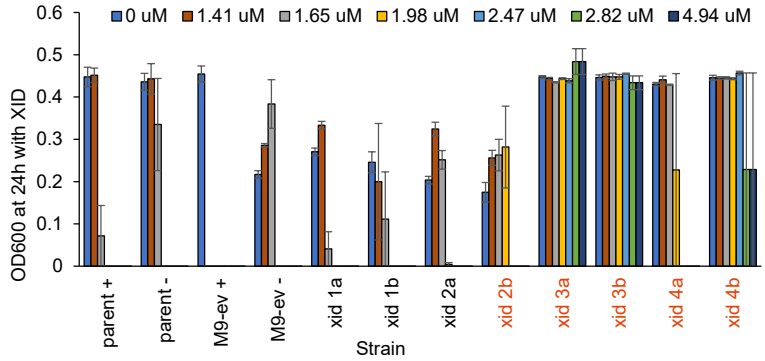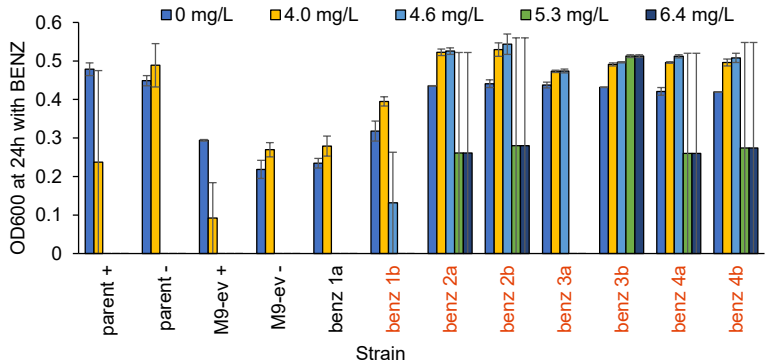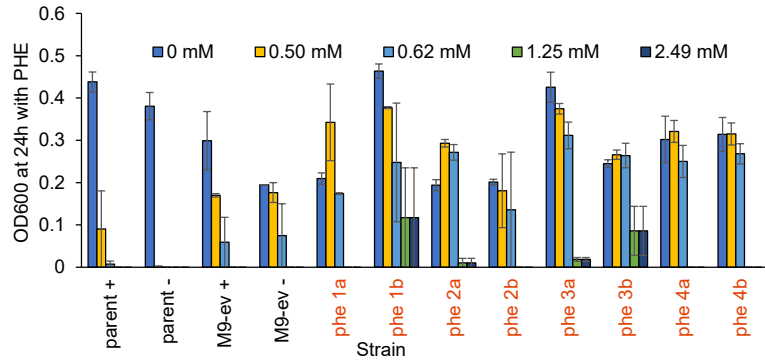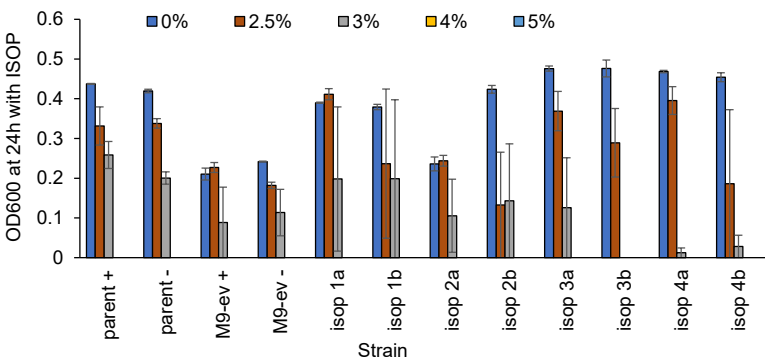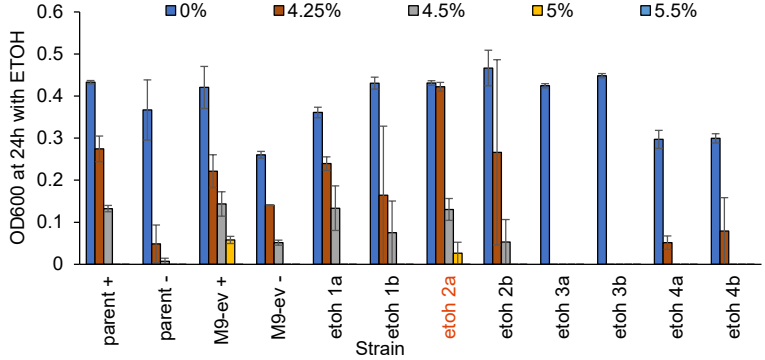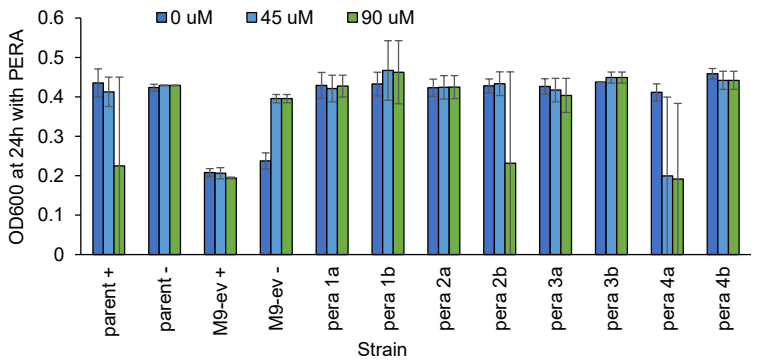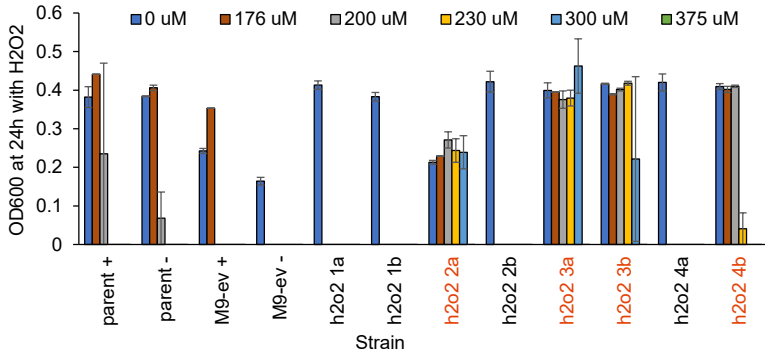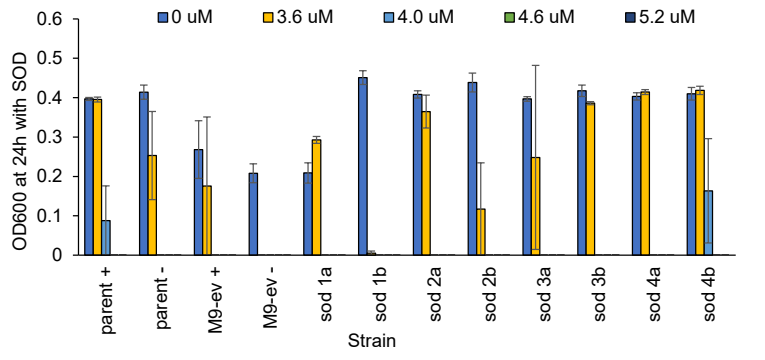

Supplement: Supplementary file 1 [file Data_Sheet_1.zip › Supplementary Figure 2.pdf]

**S3**

■ versus media-evolved strain (M9-ev+ or M9-ev-)  
 ■ versus parent strain (parent+ or parent-)

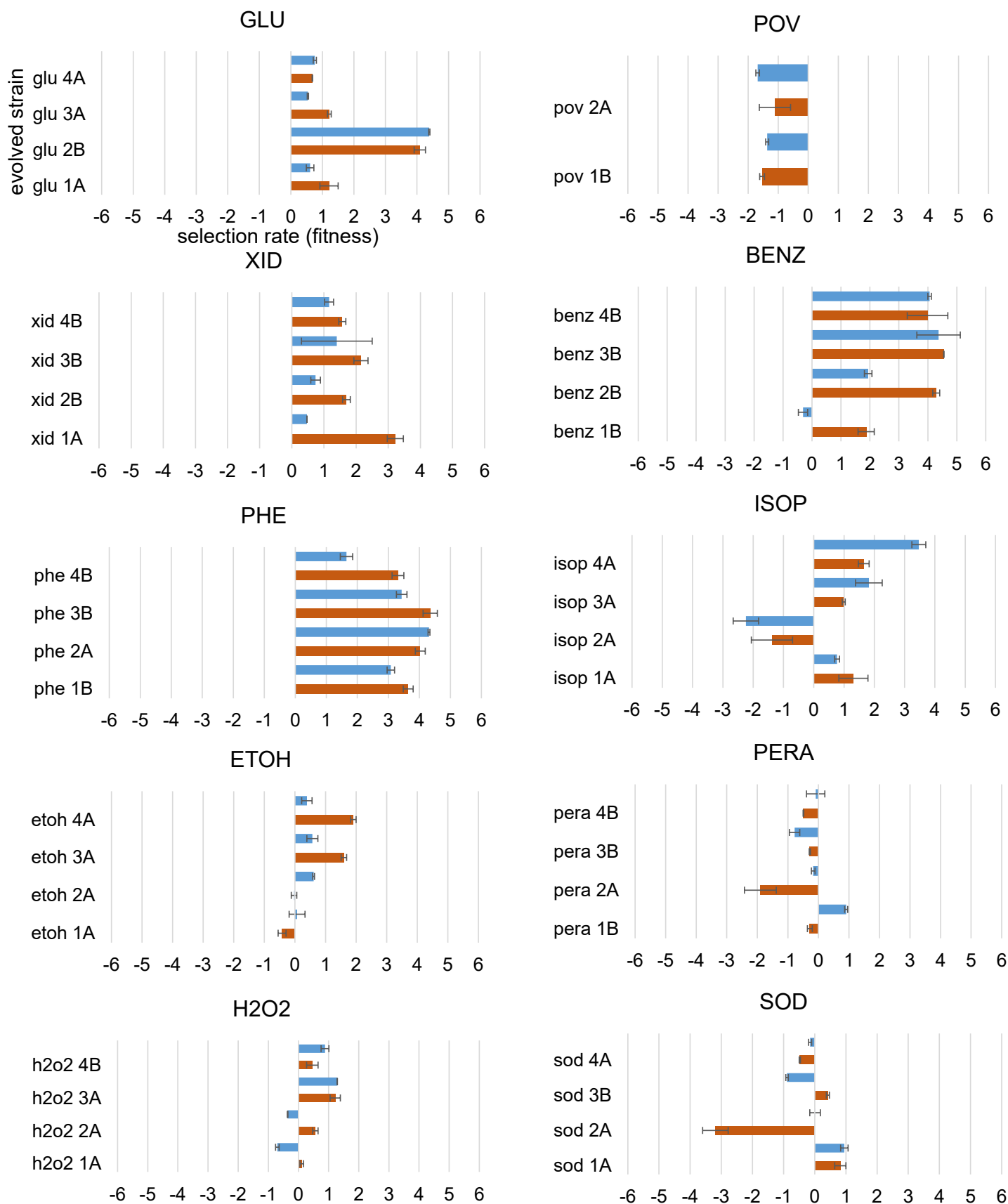

Supplement: Supplementary file 1 [file Data_Sheet_1.zip › Supplementary Figure 3.pdf]

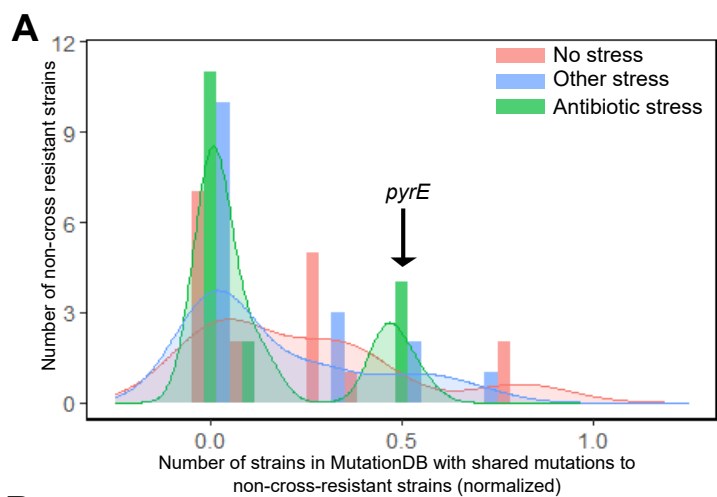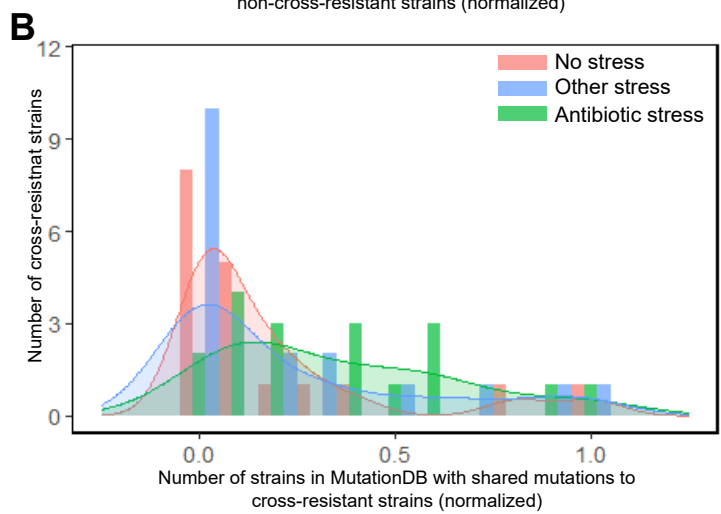

Supplement: Supplementary file 1 [file Data_Sheet_1.zip › Supplementary Figure 4.pdf]

S5

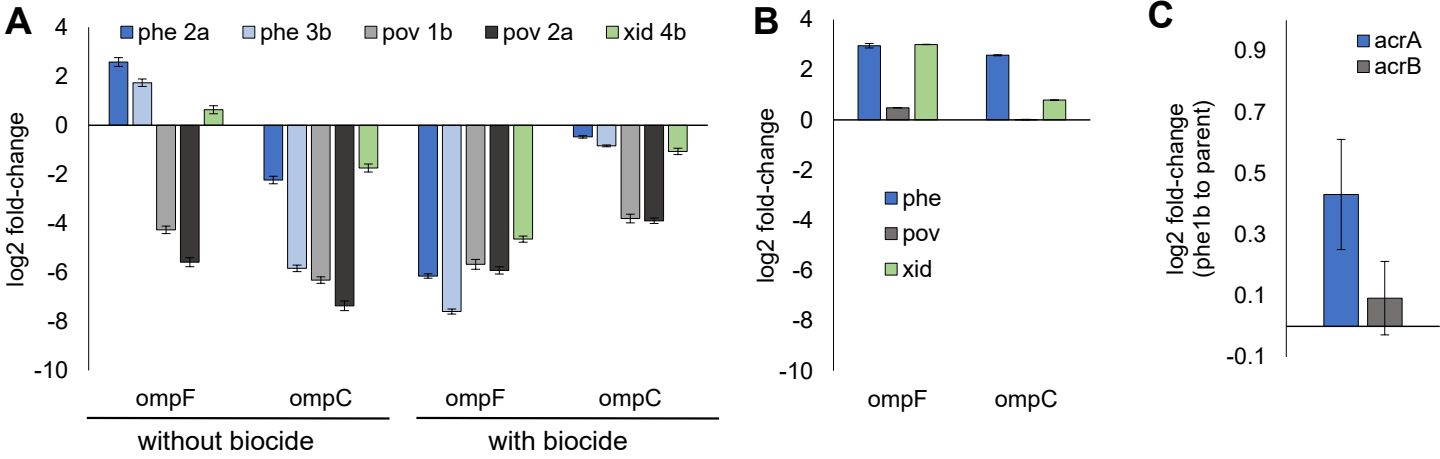

Supplement: Supplementary file 1 [file Data_Sheet_1.zip › Supplementary Figure 5.pdf]

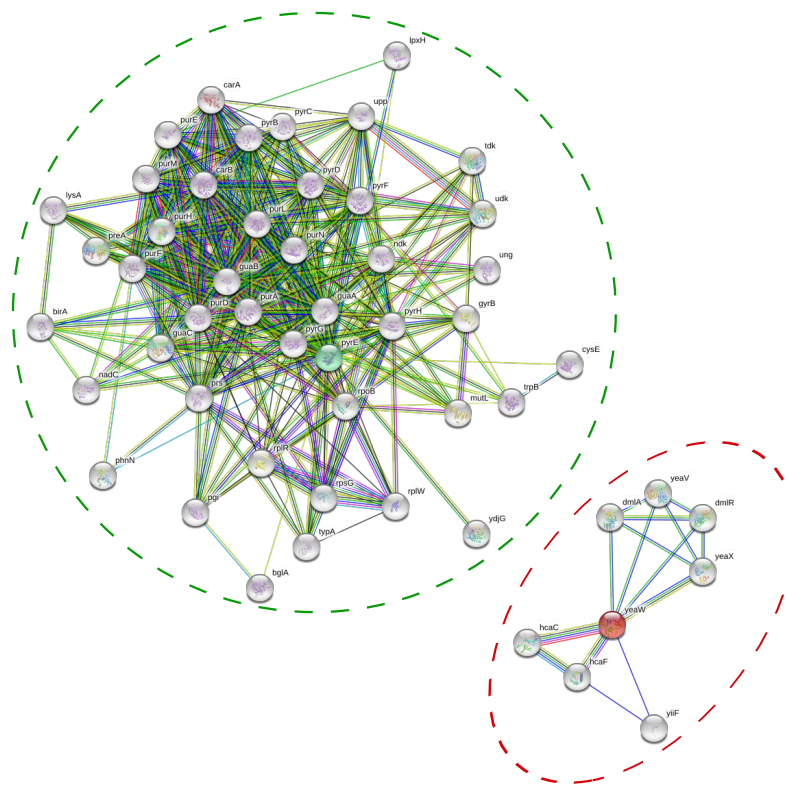

Supplement: Supplementary file 1 [file Data_Sheet_1.zip › Supplementary Figure 6.pdf]

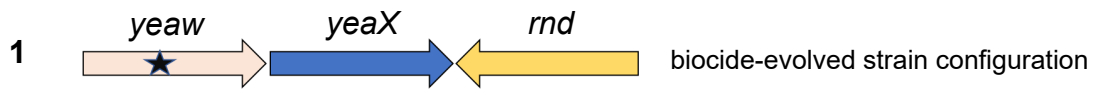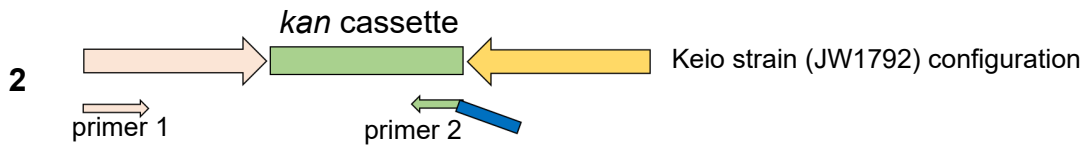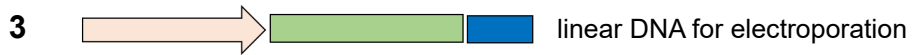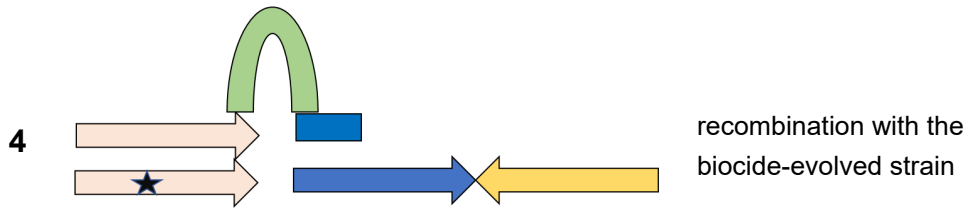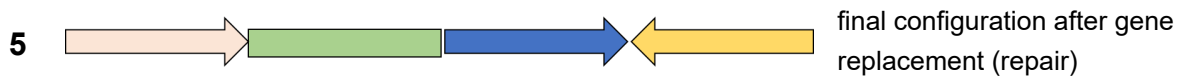

Supplement: Supplementary file 1 [file Data_Sheet_1.zip › Supplementary Figure 7.pdf]
